# A novel model of reno-cardiac syndrome in the C57BL/ 6 mouse strain

**DOI:** 10.1186/s12882-018-1155-3

**Published:** 2018-12-04

**Authors:** Julius E. Kieswich, Jianmin Chen, Samira Alliouachene, Paul W. Caton, Kieran McCafferty, Christoph Thiemermann, Muhammad M. Yaqoob

**Affiliations:** 1 Diabetic Kidney Disease Centre, Renal Unit, Barts Health NHS Trust, The Royal London Hospital, Whitechapel Road, London, E1 1BB UK; 2 Center for Translational Medicine and Therapeutics, William Harvey Research Institute, Barts and the London School of Medicine and Dentistry, Queen Mary University of London, London, EC1M 6BQ UK; 3 Diabetes Research Group, Division of Diabetes and Nutritional Sciences, King’s College London, Hodgkin Building, Guy’s Campus, London, UK

**Keywords:** Reno-cardiac syndrome, RCS, Chronic kidney disease, CKD, Cardiac hypertrophy, C57/BL 6 mouse, Cardiac dysfunction, Renal fibrosis, Experimental renal failure

## Abstract

**Background:**

The end stage renal disease population has a 20 fold higher incidence of cardiovascular mortality compared to the overall population. The development of reno-cardiac syndrome in these patients will result in cardiovascular events to be the cause of 50% of fatalities. There is therefore a need to research improved therapeutic strategies to combat renal cardiac pathologies. Murine in vivo models contribute greatly to such research allowing for specific genetic modification and reduced miscellany, however there is currently no reliable model of reno-cardiac syndrome in the most common genetically modified mouse strain, the C57BL/6. In this study we have manipulated an established model of chronic renal disease using adenine infused diet and prolonged the course of its pathology achieving chronic renal failure and subsequent reno-cardiac syndrome in the C57BL/6 mouse.

**Methods:**

Eight week-old male C57BL/ 6 mice were acclimatised for 7 days before administration of a 0.15% adenine diet or control diet for 20 weeks.

Cardiac function was assessed in mice at week 20 by echocardiography. At experiment termination blood and urine samples were analysed biochemically and organ dysfunction/injury was determined using immunoblotting and immunohistochemistry.

**Results:**

Administration of 0.15% adenine diet caused progressive renal failure resulting in reno-cardiac syndrome. At endpoint uraemia was confirmed by blood biochemistry which in the adenine fed mice showed significant increases in serum creatinine, urea, calcium (*P* < 0.0001) potassium (*P* < 0.05), and a significantly reduced glomerular filtration rate (*P* < 0.05). Reno-cardiac syndrome was confirmed by a significantly increased heart to body weight ratio (*P* < 0.0001) and echocardiography which showed significant reductions in percentage of ejection fraction, fractional shortening, fractional area change, (*P* < 0.0001) and an increase in left ventricular end diastolic volume (*P* < 0.05). Immunoblotting of kidney and heart tissue showed increased apoptosis (caspase 3) and fibrosis (fibronectin) and increases in the cardiac levels of phosphorylated Akt, and renal total Akt. Immunohistochemistry for α-SMA, collagen 1 and collagen 3 further confirmed fibrosis.

**Conclusions:**

We present a novel regimen of adenine diet which induces both chronic kidney disease and reno-cardiac syndrome in the C57/BL6 mouse strain. The non-surgical nature of this model makes it highly reproducible compared to other models currently available.

**Electronic supplementary material:**

The online version of this article (10.1186/s12882-018-1155-3) contains supplementary material, which is available to authorized users.

## Background

Chronic reno-cardiac syndrome (RCS), a branch of the general cardio renal syndrome in which impaired renal function inflicts consequential damage on to the cardiac vasculature (defined by Ronco et al. [1]) is prevalent in the end stage renal disease (ESRD) population having a 20 fold higher incidence of cardiovascular mortality (encompassing the clinical scenarios of myocardial infarction, sudden death, arrhythmia and cardiomyopathy) compared to the population as a whole [2, 3]. Approximately 50% of mortality in ESRD patients is as a result of cardiovascular events [4, 5]. These factors highlight the need for the continued improvement of therapeutic strategies to combat RCS.

The employment of animal models as tools to explore disease pathologies has greatly enhanced research especially with the development of technology to genetically modify strains. The most successful animal model of RCS is the sub-total nephrectomy model in rats where a portion (5/6ths) of the kidney mass is surgically removed or negated [6]. The resulting renal ischaemia initiates stimulation of the sympathetic nervous system, triggering the renin–angiotensin–aldosterone system and inhibiting nitric oxide synthesis eventually causing hypertension, left ventricular hypertrophy (LVH) and progressive left ventricular dilatation [7]. Fibroblast activation leads to eventual cardiac hypertrophy and fibrosis [8, 9]. Unfortunately in mice, this model is much less effective giving highly variable results [10]. This is further compounded by the fact that the C57BL/ 6 mouse strain, the preferred strain for genetic modification [11] has been shown to be resistant to developing CKD by subtotal nephrectomy [12].

Another model of CKD is a chemical method involving the addition of adenine to the diet. The peculiar metabolism of adenine compared to other purines was first described in rats by Yokozawa et al. [13] and its nephrotoxic effects were subsequently employed in the creation of a highly reproducible model of CKD in rats. However, adenine-induced CKD results in a rapid impairment of renal function and high (early) mortality, which prevents the development of RCS. In mice, the adenine model has been less successful due to their distaste of adenine causing substantial and rapid weight loss leading to mortality after 4 weeks [14]. The addition of casein into the diet masks the taste of the adenine and prolongs the model to 8 weeks [14] however this is still not long enough for cardiovascular effects to mature.

The aim of this study was to establish a non-surgical and, therefore, more replicable model of RCS in the C57BL/ 6 mouse strain. To achieve this we further manipulated the adenine diet protocol by reducing the volume of adenine in the diet to 0.15% thus, concealing the taste of the adenine (without the need for addition of casein), to the point that mice ingested it without weight loss and corresponding morbidity. In this way the diet regimen was extended to 20 weeks causing the development of CKD and subsequent RCS.

## Methods

### Animal experiments

Animal experiments were conducted in accordance with UK Home Office Animals (Scientific Procedures) Act 1986, with local ethical committee approval. 8-week-old male C57BL/ 6 mice (Charles River, Margate, UK) were maintained on a 12 h light/12 h dark cycle, receiving water ad libitum and after a 7 day acclimatisation period were randomly divided into 2 groups. One was fed a diet containing 0.15% adenine (824,534 RM1 + 0.15% Adenine; SDS diets, LBS-Biotech, Hookwood, UK, BMC Additional file 1) whilst the second group received a control diet. Diet administration continued for 20 weeks. There was no premature mortality during this period. Sample sizes (14 in the adenine treated group and 10 in the control group) were determined on the basis that the adenine treated group might show greater variability however this proved not to be the case. At the end of the experiment animals were culled under deep anaesthesia by exsanguination. Two animals from each group were sacrificed at weeks 12 and 16 in order to determine the progression of uraemia (BMC Additional file 2).

### Quantification of organ dysfunction/injury

Cardiac function was assessed in mice at week 20 by echocardiography using a Vevo-770 Imaging System (Visual Sonics, Toronto, Canada) [15]. Animals were housed in metabolic cages for 24 h for the collection of urine samples after which the experiment was terminated, and organ and blood samples were collected and sent for analysis (IDEXX Bioresearch, Ludwigsberg, Germany), for quantification of organ dysfunction/injury, (parameters and methods of measurement are provided in BMC Additional file 3).

### Immunoblotting

Immunoblotting was conducted as previously described [16] using primary antibodies against Fibronectin (Santa Cruz Biotechnology, Dallas, TX, USA), p-Akt (Ser473), total Akt, and caspase 3 (Cell Signalling Technologies, Danvers, MA, USA), Gapdh, α-smooth muscle actin, and α-tubulin (all Sigma Aldrich, Dorset, UK). Primary antibodies were used at the dilutions recommended by the manufacturer. Secondary antibodies were anti- mouse or anti rabbit (Santa Cruz Biotechnology, Dallas, USA). Immunoblotting against fibronectin was performed under non-denaturing conditions. Blinded Semi quantitative Western blot analyses were carried out in mouse heart and kidney tissues using Image J software [17].

### Immuno histochemical analysis of mouse heart, kidney and aortic sections

Following perfusion with saline, hearts, kidneys and aortas were fixed in buffered formalin, embedded in paraffin, and sectioned according to standard procedures. All tissues were stained with hematoxylin and eosin, α-smooth muscle actin, Sirius red, F4/80, CD45, collagen 1 and collagen 3, (all Abcam, Cambridge, UK). Images were captured at 20x magnification, using a Panoramic Scanning 250 microscope and analysed blindly using Panoramic Viewer software (3D Histech Ltd., Budapest, Hungary). Five images of tissues were captured per mouse and staining was quantified as percentage of total area according to published methods using Image J software [17].

### Statistical analyses

Statistical analysis was performed using GraphPad Prism 5 Software (GraphPad Software Inc., CA, US). Values are presented as mean ± SEM of n observations. Differences between adenine-treated mice and controls were calculated using the unpaired t- test. *P* < 0.05 was considered to be statistically significant.

## Results

### Bodyweight

**Fig. 1:**
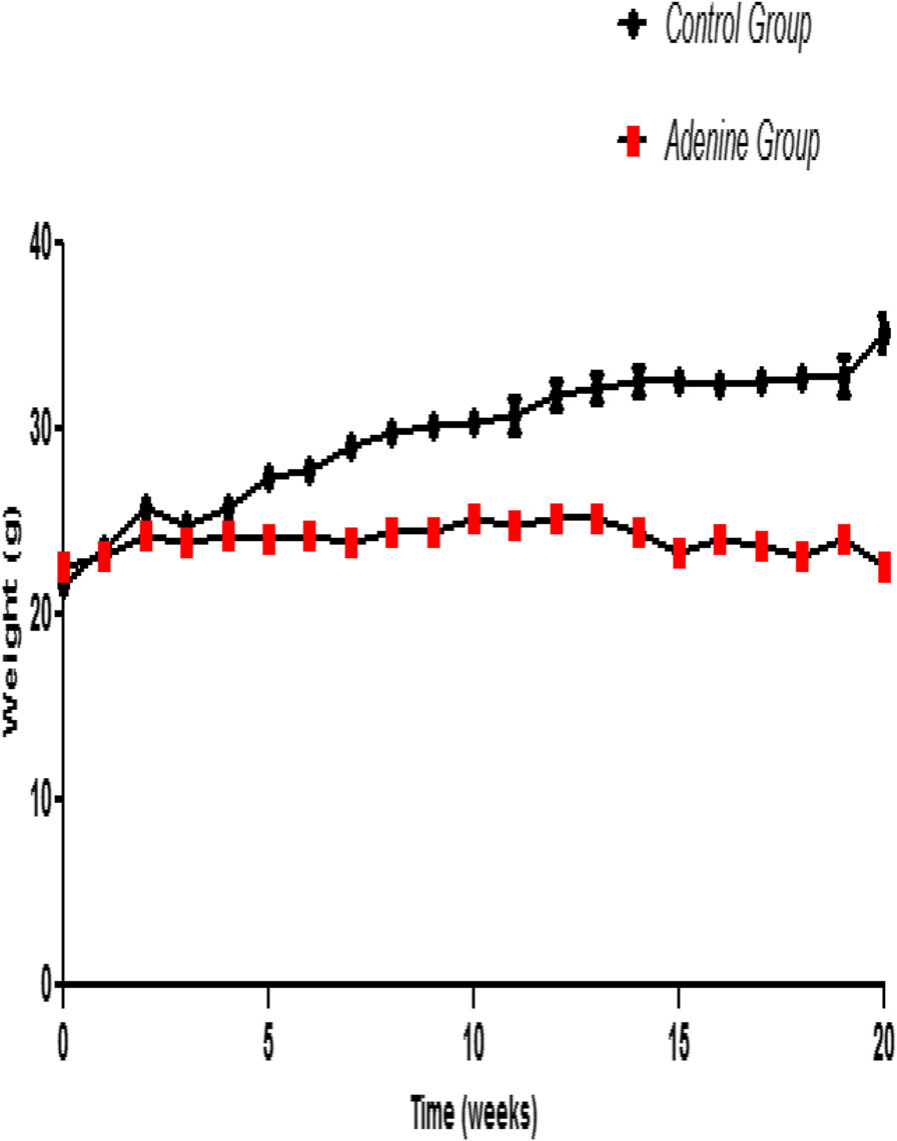
Bodyweight. Control group (*n* = 6) received standard chow for 20 weeks. Adenine treated group (*n* = 10) received standard chow with the addition of 0.15% adenine for 20 weeks. Bodyweight of adenine treated mice was maintained throughout the experimental period (20 weeks)

Changes in bodyweight over the 20 week period are depicted in Fig. 1. Unlike other adenine diet regimens, there is no initial loss of bodyweight in these mice and bodyweight is maintained until the endpoint of the study at week 20.

### Characterisation of renal dysfunction in mice administered 0.15% adenine diet for 20 weeks

Compared with animals receiving normal chow, a 20 -week diet of 0.15% adenine caused significant increases in serum and urinary markers of renal dysfunction specifically significantly higher plasma urea (*P* < 0.0001) creatinine (*P* < 0.0001), calcium (*P* < 0.0001), and potassium (*P* < 0.05) concentrations. There was no difference in serum sodium nor inorganic phosphorous between the groups. Adenine treated animals were significantly polyuric (*P* < 0.0001). The glomerular filtration rate (GFR) assessed by creatinine clearance was significantly reduced (P < 0.05), whereas the fractional excretion of sodium and potassium were significantly increased (*P* < 0.0001) [Table 1, and BMC Additional file 4].

**Table 1:** Biochemistry showing renal dysfunction

Parameter	Control	Adenine
No.	6	10
Serum urea, mmol/L	8.867 ± 0.4240	81.57 ± 1.809 ***
Serum creatinine, μmol/L	16.83 ± 3.554	114.5 ± 5.909 ***
Serum calcium, mmol/L	1.983 ± 0.01667	2.760 ± 0.08055 ***
Serum sodium, mmol/L	154.3 ± 1.333	156.5 ± 0.7491
Serum potassium, mmol/L	5.430 ± 0.1762	6.019 ± 0.07275 *
Serum i. phosphate, mmol/L	2.550 ± 0.08466	2.750 ± 0.1455
Urine flow ml/min	0.0003518 ± 0.00008339	0.002882 ± 0.0002918 ***
Urine sodium, mmol/L	218.3 ± 27.03	59.06 ± 3.652 ***
Urine potassium, mmol/L	293.9 ± 59.58	100.3 ± 8.498 ***
FE sodium, %	8208 ± 1883	99,690 ± 9764 ***
FE potassium, %	424.1 ± 127.3	6223 ± 384.0 ***
GFR, ml/min	0.1642 ± 0.04598	0.02869 ± 0.003854 *

Control group (*n* = 6) received standard chow for 20 weeks. Adenine treated group (n = 10) received standard chow with the addition of 0.15% adenine for 20 weeks. Compared with animals receiving normal chow, a 20-week diet of 0.15% adenine caused significant increases in plasma urea (*P* < 0.0001), creatinine (*P* < 0.0001), calcium (*P* < 0.0001), and potassium (P < 0.05) concentrations. Adenine treated animals were significantly polyuric (*P* < 0.0001). The glomerular filtration rate (GFR) assessed by creatinine clearance was significantly reduced (P < 0.05), whereas the fractional excretion of sodium and potassium were significantly increased (*P* < 0.0001), mean ± SEM unpaired t- test

***P < 0.0001

*P < 0.05

### Characterisation of cardiac dysfunction through echocardiography

Echocardiography showed slight, but significant, reductions in percentage of ejection fraction (EF), fractional shortening, and fractional area change (*P* < 0.0001) and, hence, an impairment of systolic contractility. There was no increase in interventricular septum thickness (IVS) in adenine treated mice, but an increase in left ventricular dimensions (left ventricular internal diastolic dimension, and left ventricular end diastolic volume; *P* < 0.05), and a significantly greater heart weight-to body weight ratio (*P* < 0.0001) [Table 2].

**Table 2:** Cardiac dysfunction

Parameter	Control	Adenine
No.	6	10
Ejection fraction, %	72.84 ± 1.425	65.14 ± 0.8203 ***
Fractional shortening, %	41.17 ± 1.092	35.41 ± 0.6011 ***
Fractional area change, %	49.82 ± 0.6524	42.80 ± 1.149 ***
LVID, mm	3.832 ± 0.1192	4.257 ± 0.04804 *
IVS, mm	0.9647 ± 0.08316	1.039 ± 0.04658
Heart/bodyweight ratio	0.004341 ± 0.0001352	0.006031 ± 0.0001553 ***

Control group (*n* = 6) received standard chow for 20 weeks. Adenine treated group (*n* = 10) received standard chow with the addition of 0.15% adenine for 20 weeks. Echocardiography showed slight, but significant, reductions in percentage of ejection fraction, fractional shortening, and fractional area change (*P* < 0.0001). There was an increase in left ventricular dimensions (left ventricular internal diastolic dimension (LVID) and left ventricular end Diastolic volume; *P* < 0.05), and a significantly greater heart weight-to body weight ratio (*P* < 0.0001), mean ± SEM unpaired t- test

***P < 0.0001

*P < 0.05

### Effects of 20 week 0.15% adenine diet administration on the expression of Akt and phosphorylation of Akt in hearts and kidneys of mice

**Fig. 2:**
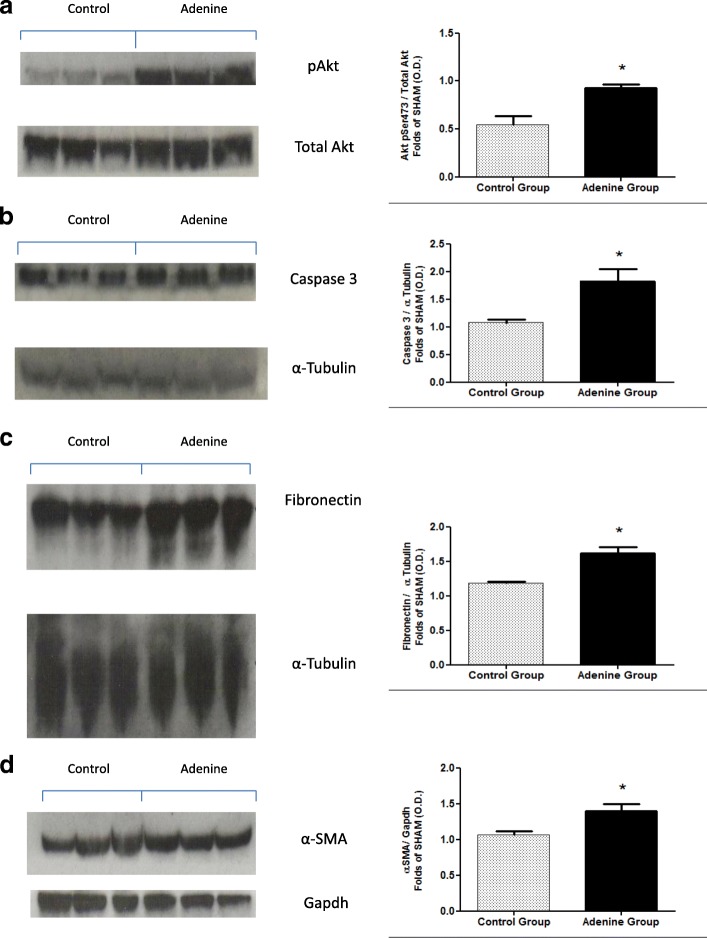
Cardiac expression of phospho –Akt, caspase 3, fibronectin and α-SMA. Control group (*n* = 6) received standard chow for 20 weeks. Adenine treated group (*n* = 10) received standard chow with the addition of 0.15% adenine for 20 weeks. Representational Western blots (*n* = 3). Compared with mice on a normal diet, adenine fed mice had a significantly higher degree of cardiac phosphorylation of AKT on Ser473 (**a**) (*P* < 0.05), significantly increased expression of caspase 3 (**b**) (P < 0.05), a significant increase in the expression of fibronectin (**c**) (*P* < 0.05), and a significant increase in the expression of α-smooth muscle actin (**d**) (*P* < 0.05), mean ± SEM unpaired t- test. (NB: Original blots ‘BMC Additional file 7)

**Fig. 3:**
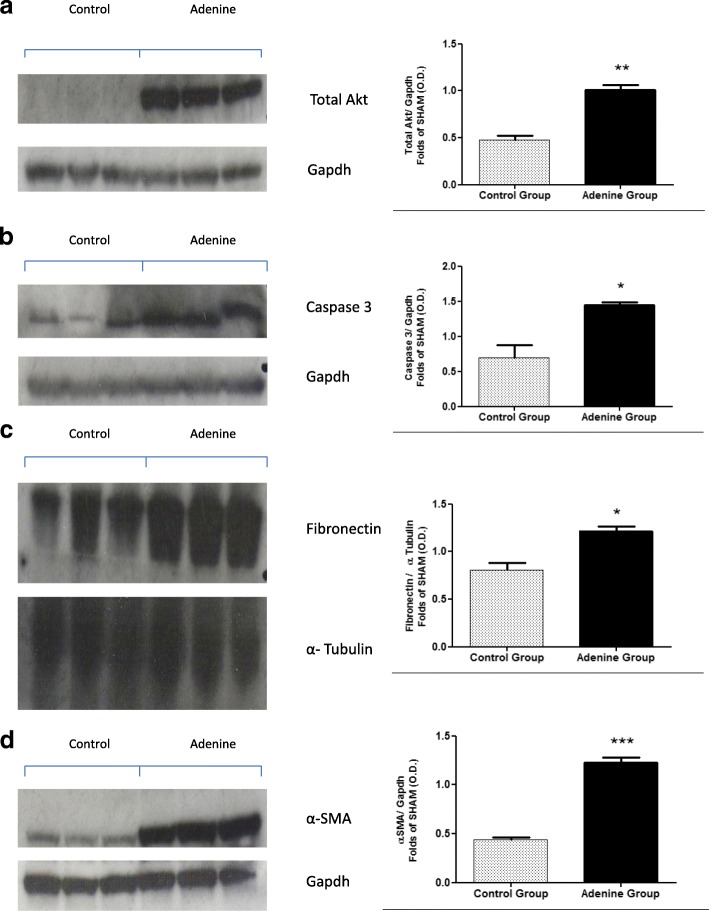
Renal expression of Total Akt, caspase 3, fibronectin and α-SMA. Control group (*n* = 6) received standard chow for 20 weeks. Adenine treated group (*n* = 10) received standard chow with the addition of 0.15% adenine for 20 weeks. Representational Western blots (*n* = 3). Compared with mice on a normal diet, adenine fed mice had a significantly higher expression of renal total Akt (**a**) (*P* < 0.05), significantly increased expression of caspase 3 (**b**) (*P* < 0.05), a significant increase in the expression of fibronectin (**c**) (*P* < 0.05), and a significant increase in the expression of α-smooth muscle actin (**d**) (*P* < 0.0001), mean ± SEM unpaired t- test. (NB: Original blots ‘BMC Additional file 8’)

To gain a better insight into the mechanisms underlying both cardiac and vascular dysfunction in mice after 20 weeks of 0.15% adenine diet, we investigated signalling events in the hearts and kidneys of these mice. Compared with mice on a normal diet, adenine fed mice had a significantly higher degree of cardiac phosphorylation of Akt on Ser473 (*P* < 0.05), [Fig. 2, (a)], and a significantly higher expression of renal total Akt (*P* < 0.01), [Fig. 3, (a)].

### Effects of 20 week 0.15% adenine diet administration on apoptosis in hearts and kidneys of mice

Apoptosis has been shown to be prevalent during heart disease both in the heart and vasculature [18, 19]. Here we investigated the effects of prolonged administration of 0.15% adenine diet on cardiac and renal apoptosis. Compared with mice on a normal diet adenine fed mice had significantly increased expression of caspase 3 indicating apoptosis in these tissues (*P* < 0.05), [Fig. 2, (b) and Fig. 3, (b)].

### Effects of 20 week 0.15% adenine diet administration on fibrosis in hearts and kidneys of mice

Compared with mice on a normal diet, adenine fed mice showed significant increases in the expression of fibronectin in both the heart and kidneys (*P* < 0.05), [Fig. 2, (c) and Fig. 3, (c)] and in α-smooth muscle actin in the hearts (*P* < 0.05), [Fig. 2, (d) and kidneys (*P* < 0.0001), [Fig. 3 (d)].

**Fig. 4:**
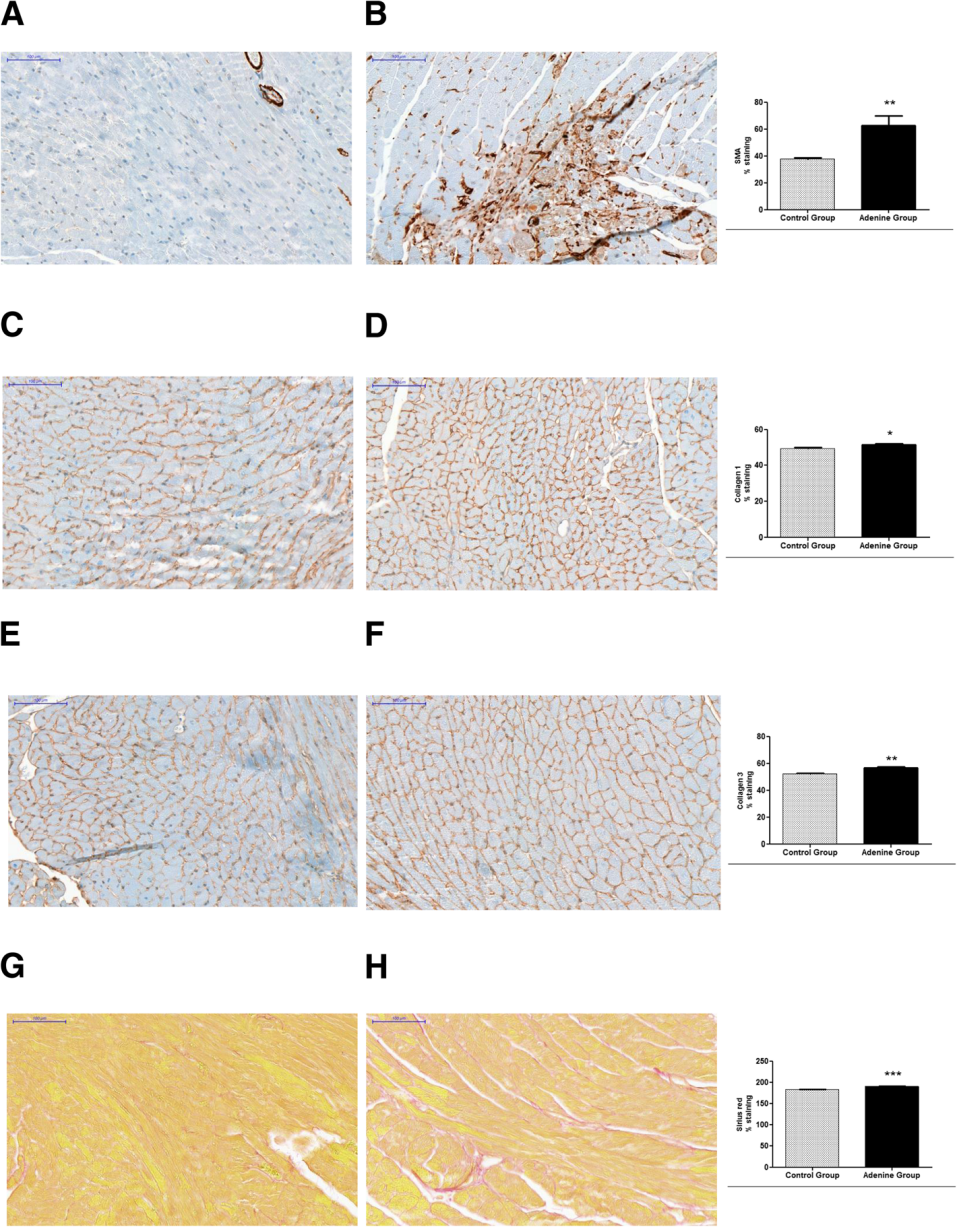
Evidence for cardiac hypertrophy through staining with sirius red and immunostaining for α-SMA, collagen 1, and collagen 3. Control group (*n* = 6) received standard chow for 20 weeks. Adenine treated group (*n* = 10) received standard chow with the addition of 0.15% adenine for 20 weeks. Immunostaining of hearts showed a significant increase in α-SMA staining of adenine group (**b**) compared to control group (**a**) (*P* < 0.01). A significant increase in collagen 1 staining of adenine group (**d**) compared to control group (**c**) (*P* < 0.05). A significant increase in collagen 3 staining of adenine group (**f**) compared to control group (**e**) (*P* < 0.01), and a significant increase in sirius red staining of adenine group (**h**) compared to control group (**g**) (*P* < 0.0001), mean ± SEM unpaired t- test. Representational images (3 mice per group)

**Fig. 5:**
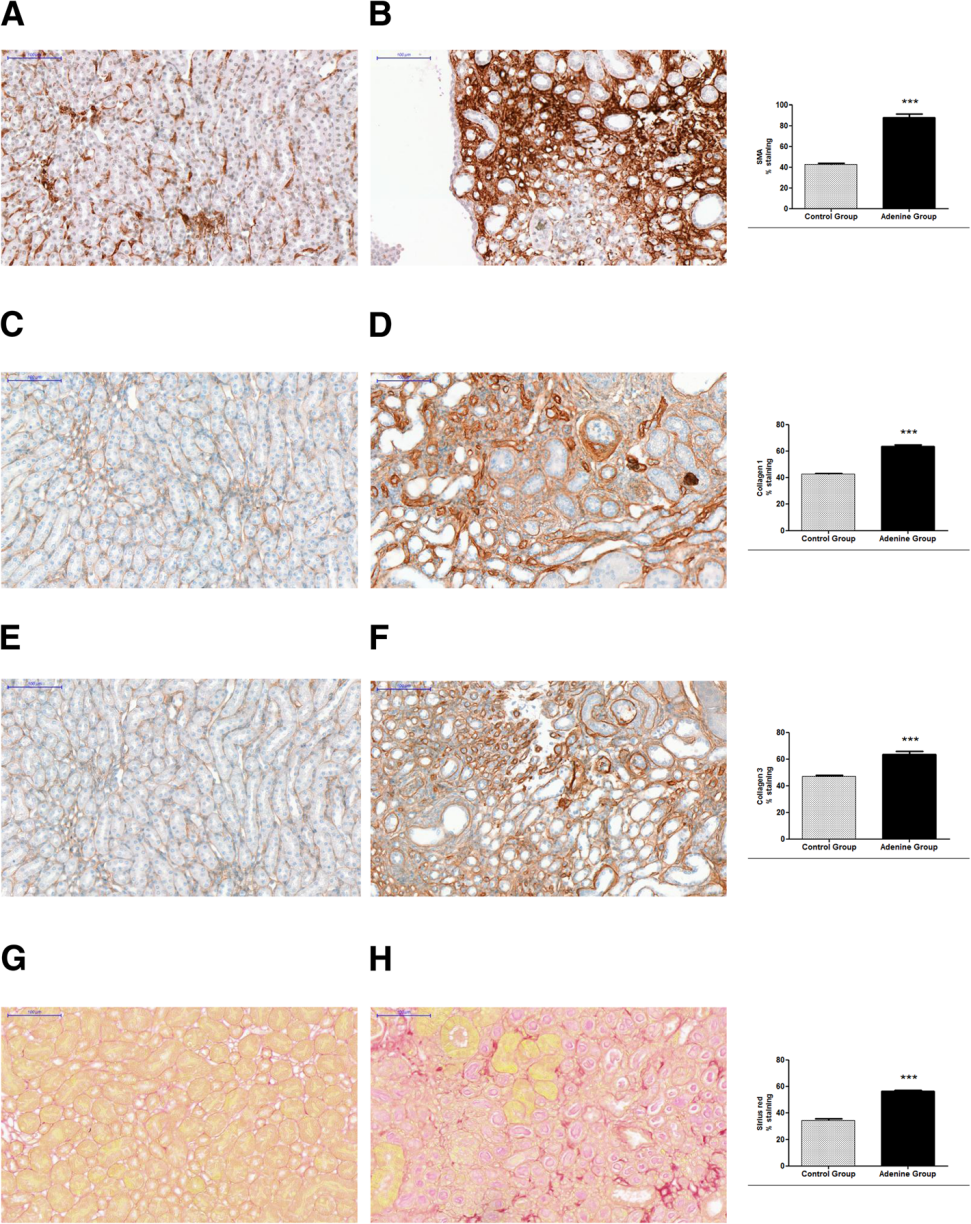
Evidence for renal hypertrophy through staining with sirius red and immunostaining for α-SMA, collagen 1, and collagen 3. Control group (*n* = 6) received standard chow for 20 weeks. Adenine treated group (*n* = 10) received standard chow with the addition of 0.15% adenine for 20 weeks. Immunostaining of kidneys showed a significant increase in α-SMA staining of adenine group (**b**) compared to control group (**a**) (*P* < 0.0001). A significant increase in collagen 1 staining of adenine group (**d**) compared to control group (**c**) (*P* < 0.0001). A significant increase in collagen 3 staining of adenine group (**f**) compared to control group (**e**) (*P* < 0.0001), and a significant increase in sirius red staining of adenine group (**h**) compared to control group (**g**) (*P* < 0.0001), mean ± SEM unpaired t- test. Representational images (3 mice per group)

Immunostaining of hearts showed significant increases in anti α-SMA (*P* < 0.01), collagen 1 (P < 0.05), collagen 3 (*P* < 0.01), and sirius red (*P* < 0.0001), of adenine treated mice compared to controls [Fig. 4 and BMC Additional file 5]. Immunostaining of kidneys showed significant increases in anti α-SMA (*P* < 0.0001), collagen 1 (*P* < 0.0001), collagen 3 (*P* < 0.0001), and sirius red (*P* < 0.0001), of adenine treated mice compared to controls [Fig. 5 and BMC Additional file 5].

### Effects of 20 week 0.15% adenine diet administration on inflammation in hearts, kidneys and aortas of mice

**Fig. 6:**
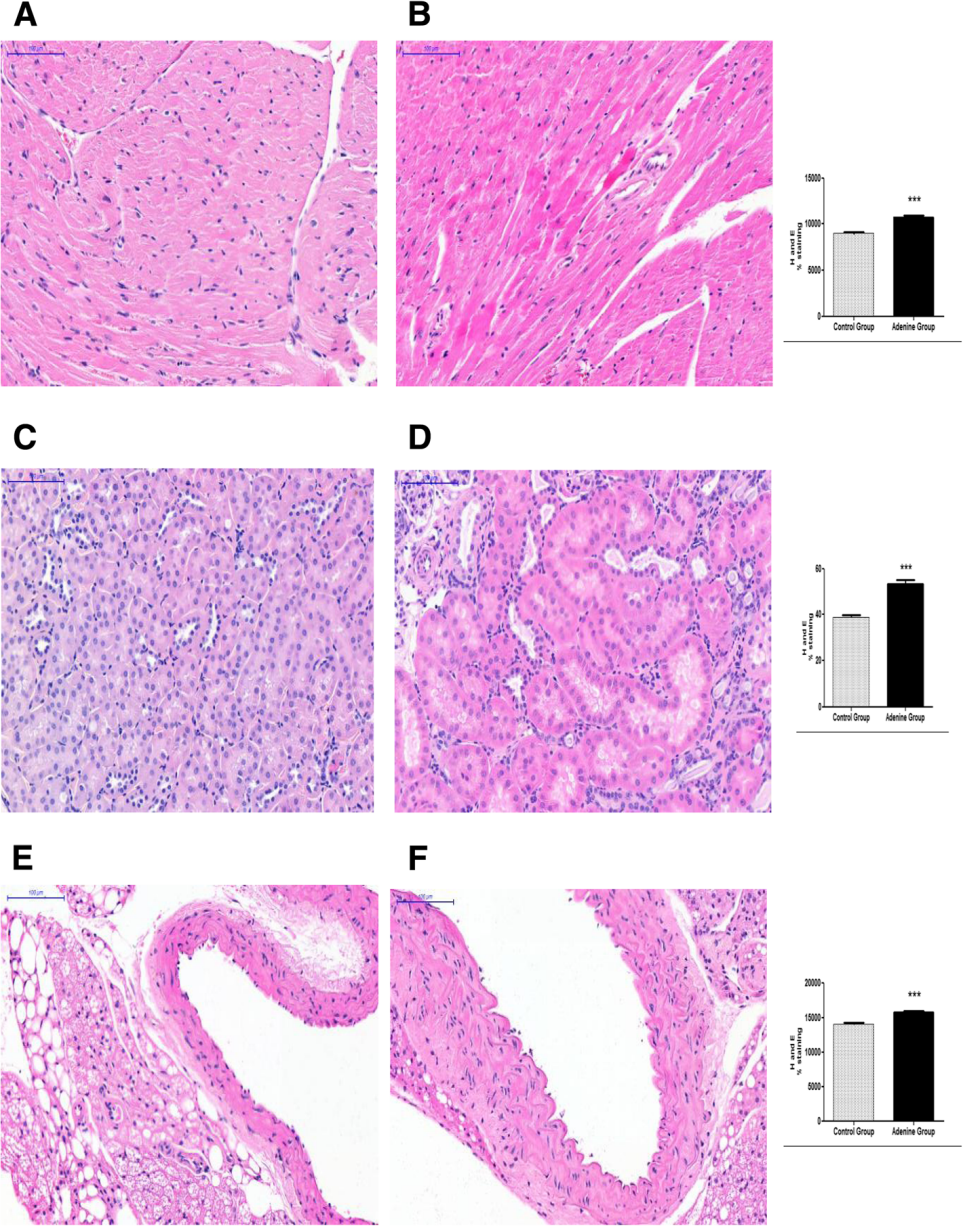
Evidence for inflammation through haemotoxylin and eosin staining. Control group (*n* = 6) received standard chow for 20 weeks. Adenine treated group (*n* = 10) received standard chow with the addition of 0.15% adenine for 20 weeks. H and E staining of hearts showed a significant increase in the adenine group (**b**) compared to control group (**a**) (*P* < 0.0001). H and E staining of Kidneys showed a significant increase in the adenine group (**d**) compared to control group (**c**) (*P* < 0.0001). H and E staining of aortas showed a significant increase in the adenine group (**f**) compared to control group (**e**) (*P* < 0.0001), mean ± SEM unpaired t- test. Representational images (3 mice per group)

**Fig. 7:**
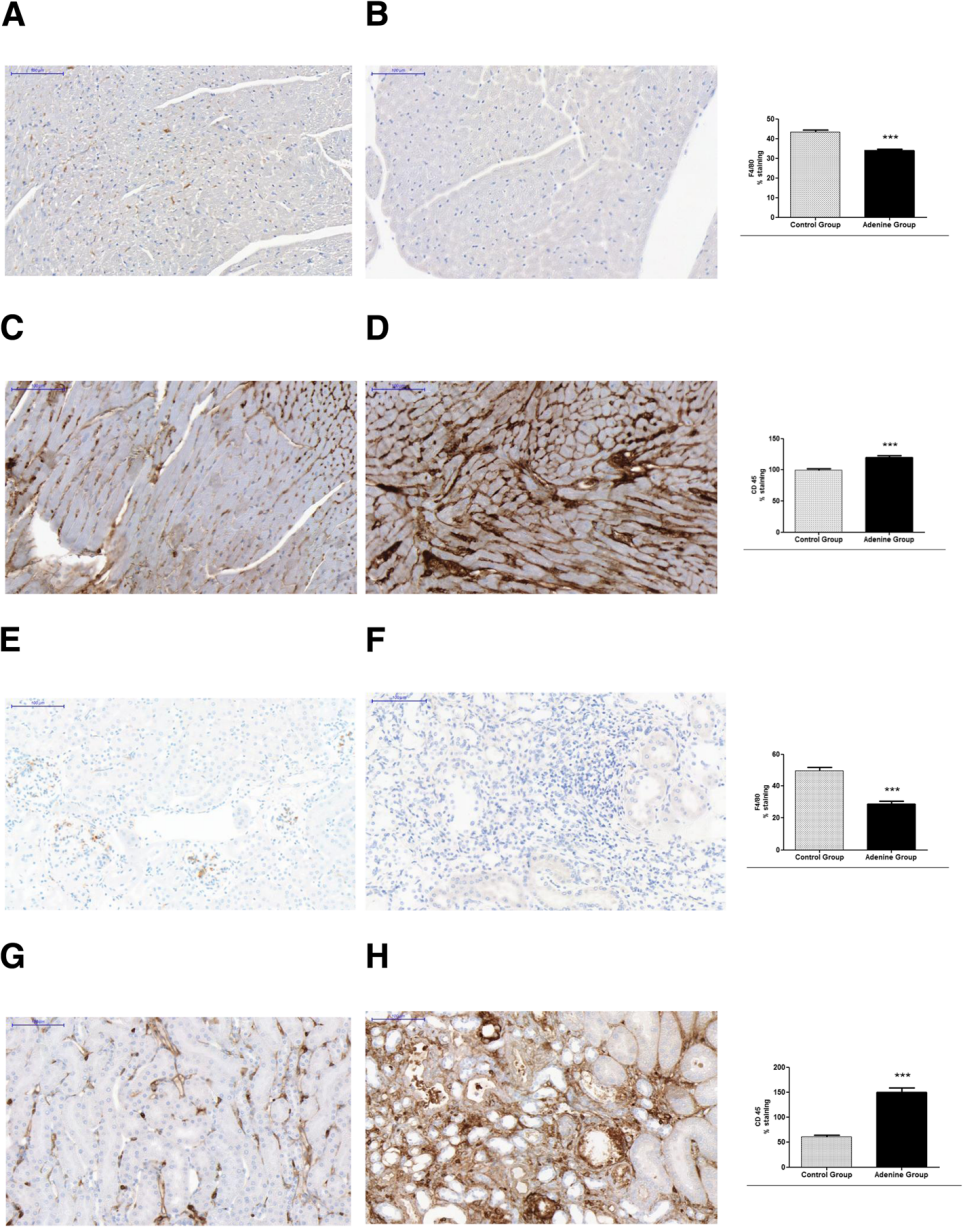
Evidence for cardiac and renal inflammation through immunostaining for F4/80 and CD45. Control group (*n* = 6) received standard chow for 20 weeks. Adenine treated group (*n* = 10) received standard chow with the addition of 0.15% adenine for 20 weeks. Immunostaining of hearts and kidneys showed a significant decrease in F480 staining of adenine group (**b**, hearts and **f**, kidneys) compared to control group (**a**, hearts and **e**, kidneys) (*P* < 0.0001). Immunostaining of hearts and kidneys for CD45 showed a significant increase in staining of adenine group (**d**, hearts and **h**, kidneys) compared to control group (**c**, hearts and **g**, kidneys) (*P* < 0.0001), mean ± SEM unpaired t- test. Representational images (3 mice per group)

Haemotoxylin and eosin staining was significantly increased in hearts, kidneys and aortas of adenine treated mice compared to normal chow treated indicating an increased degree of inflammation (*P* < 0.0001), [Fig. 6, and BMC Additional file 6]. This was further confirmed by F4/80 stain which was significantly decreased in adenine treated hearts and kidneys indicating activation of macrophages in these tissues compared to controls [20] (*P* < 0.0001), [Fig. 7 and BMC Additional file 6].

Immunostaining of hearts and kidneys for CD45 showed significant increases in adenine treated animals compared to controls (*P* < 0.0001), [Fig. 7 and BMC Additional file 6], also indicating leucocyte infiltration.

## Discussion

Translational medicine requires the ability to investigate the in vivo expression of pathological genes in RCS thus necessitating the development of a reliable model of CKD in the C57BL/ 6 mouse the most common background strain for transgenic and knockout breeds.

Constraining factors in currently available murine models of RCS include high variability due to limitations of surgical skill and high mortality due to the intolerance of mice to such acute surgical intervention [21–25]. This is further confounded by the apparent resistance of the C57BL/ 6 strain to surgically induced uraemia [12].

The induction of CKD by the administration of a diet containing high adenine content has been successfully employed in rat studies of renal failure [26]. The metabolic product of adenine is 2,8-dihydroxyadenine which forms a precipitate of crystals in the proximal tubule specifically in the microvilli and apical epithelial region causing inhibition of tubular function and resulting in hyperphosphatemia, secondary hyperparathyroidism, bone disease and vascular calcification [27, 28].

A model based on diet administration as opposed to surgical procedure would obviously alleviate operator variability however the use of adenine diet to induce CKD in mice has been unsuccessful due to their aversion to the taste of adenine resulting in malnutrition based morbidity and mortality. Although manipulations of the adenine regimen have met with some success, the requirement of prolonging the model long enough to induce RCS has not yet been achieved. To meet this necessity we have investigated the effects of administration of a less detectable low dose of adenine diet over an extended period of 20 weeks.

Our results showed that the mice tolerated the diet well with no weight loss throughout the experimental period. Unlike surgical models of CKD there was no premature mortality and endpoint serum and urine biochemistry showed that all animals developed a similar degree of CKD.

Interestingly, unlike other adenine regimens, serum creatinine was significantly increased in CKD mice indicating no sign of malnourishment and also unlike other adenine regimens, the fact that the diet was given at a constant dose throughout meant that there was no requirement to monitor serum parameters during the course of the experiment in order to adjust the dosage accordingly.

Echocardiography suggested a small, but significant, impairment in systolic function (EF) in mice with adenine diet. However echocardiography was unable to detect an increased interventricular septum thickness in the adenine group but rather an increase in left ventricular dimensions (left ventricular internal diastolic dimension or left ventricular end diastolic volume, LVID). This taken together with a significantly greater heart weight-to body weight ratio (a surrogate marker for myocardial hypertrophy [29]), could imply development of an eccentric hypertrophy of the left ventricle (dilation of left ventricular chamber coupled with mild to moderate increased wall thickness [30]). Up to 50% of patients with heart failure have diastolic dysfunction with relatively well-preserved EF, which is termed “heart failure with preserved ejection fraction (HFpEF)” [31]. Due to the mild decrease in EF in mice with adenine diet, it is possible that this model can be characterised as HFpEF, however future study of diastolic function in this model is needed to confirm this.

We did not measure blood pressure in these mice however we have previously reported a lower degree of hypertension in adenine induced uremic animals when compared to sub-total nephrectomised animals despite a greater level of renal failure in the former [32].

RCS was further confirmed by a significantly higher degree of cardiac phosphorylation of Akt suggesting upregulation of cell survival mechanisms in response to injury [33], and evidence of continuing detrimental effects on the heart [34, 35]. H and E, CD45 and F4/80 staining reinforced evidence of inflammation. There was also an increased expression of the fibrotic biomarkers fibronectin, α-SMA, sirius red and collagens 1 and 3 in the intermuscular spaces of the hearts indicating increased myofibroblast formation [36, 37] possibly due to increased pre or afterload hypertrophy [38].

Fibrosis in the kidneys was confirmed with the same biomarkers and by a higher expression of renal total Akt, an important regulator of epithelial-mesenchymal transition of tubular epithelial cells into a myofibroblast phenotype, another indicator of compensatory renal hypertrophy [39–41].

Overall these results imply that our method has been effective in the induction of CKD and RCS in the C57BL/ 6 mouse strain. It’s main advantage over surgical models being its reduction in disparity between operators thus lowering animal volume required per experiment and also eliminating the necessity for surgical expertise and equipment. Its main advantage over other adenine based regimens being its simplicity and longevity through decreased morbidity.

We acknowledge that other nonsurgical models of RCS are already in existence and deserve comment. The administration of carbon tetrachloride is an established method of causing simultaneous cardiac and renal dysfunction and has been successfully employed in the C57BL/ 6 strain [42]. However this method is known to initiate undesirable injury in other organs [43]. This is also the case with other forms of chemical nephrectomy where the physiological effects of agents such as cisplatin, uranyl nitrate or adriamycin on the whole body system are impossible to supervise [44–46].

Obviously we recognise the fact that a chemical nephrectomy model such as adenine administration has disadvantages. As mentioned by Jia et al. [14], an adenine model is based on adenine metabolites causing tubular toxicity and therefore a tubular-interstitial disease rather than glomerular scarring secondary to vascular damage which is the most familiar source of human CKD. However, even in human CKD, tubular interstitial fibrosis is the best predictor of renal failure irrespective of the aetiology of the renal insult. Accepting these facts we realise the limitations of our method of trying to physiologically mimic the complete RCS scenario however such limitations apply to many established disease models.

## Conclusions

There is no one, perfect animal model that can completely simulate the intricacy of RCS however the prolonged administration of 0.15% adenine diet which we here propose overcomes many of the disadvantages presented by currently employed animal models. And the potency of this model in the C57BL/ 6 mouse allows for it to be utilised in the majority of genetically modified mouse strains.

## Additional files

Additional file 1: Adenine diet composition. (PDF 1108 kb)

Additional file 2: Weeks 12 [a] and 16 [b] serum biochemistry data. Two animals from each group were sacrificed at weeks 12 and 16 in order to determine the progression of uraemia. Serum urea and creatinine were determined by an enzymatic method (IDEXX Bioresearch, Ludwigsberg, Germany). (PDF 95 kb)

Additional file 3: **Table S3.** Biochemistry measurements methods. (PDF 168 kb)

Additional file 4: Raw datasets. (XLSX 66 kb)

Additional file 5: All slides, collagen 1, (control [a], adenine [e]). Collagen 3, (control [b], adenine [f]). α-SMA (control [c], adenine [g]). Sirius red, (control [d], adenine [h]). (PDF 370 kb)

Additional file 6: All slides, H and E, (control [a], adenine [d]). CD45, (control [b], adenine [e]). F4/80, (control [c], adenine [f]). (PDF 323 kb)

Additional file 7: Uncropped Western blots: hearts p AKT/total AKT ([a]/[b]), hearts caspase 3/α-tubulin ([c]/[d]), hearts α-SMA/Gapdh ([e]/[f]), hearts fibronectin/α-tubulin ([g]/[h]). (PDF 207 kb)

Additional file 8: Uncropped Western blots: kidneys total AKT/Gapdh ([a]/[b]), kidneys fibronectin/α-tubulin, ([c]/[d]), kidneys caspase 3/Gapdh ([e]/[f]), kidneys α-SMA/Gapdh ([g]/[h]). (PDF 181 kb)

## Abbreviations

CKD: Chronic kidney disease
EF: Ejection fraction
ESRD: End stage renal disease
HFpEF: Heart failure with preserved ejection fraction
IVS: Interventricular septum thickness
LVID: Left ventricular internal diastolic dimension
RCS: Reno-cardiac syndrome
SMA: Smooth muscle actin

## References

[1] Ronco C, Haapio M, House AA, Anavekar N, Bellomo R (2008). Cardiorenal syndrome. J Am Coll Cardiol.

[2] Go AS, Chertow GM, Fan D, McCulloch CE, Hsu CY (2004). Chronic kidney disease and the risks of death, cardiovascular events, and hospitalization. N Engl J Med.

[3] Ross L, Banerjee D (2013). Cardiovascular complications of chronic kidney disease. Int J Clin Pract.

[4] de Jager DJ, Grootendorst DC, Jager KJ, van Dijk PC, Tomas LM, Ansell D, Collart F, Finne P, Heaf JG, De Meester J (2009). Cardiovascular and noncardiovascular mortality among patients starting dialysis. JAMA.

[5] Steenkamp R, Shaw C, Feest TUK (2013). Renal registry 15th annual report: chapter 5 survival and causes of death of UK adult patients on renal replacement therapy in 2011: national and centre-specific analyses. Nephron Clin Pract.

[6] Morrison AB (1962). Experimentally induced chronic renal insufficiency in the rat. Lab Investig.

[7] Kumar S, Bogle R, Banerjee D (2014). Why do young people with chronic kidney disease die early?. World J Nephrol.

[8] Hewitson TD. Fibrosis in the kidney: is a problem shared a problem halved? Fibrogenesis Tissue Repair 2012 5(Suppl 1):S14.10.1186/1755-1536-5-S1-S14. 10.1186/1755-1536-5-S1-S14 PMC3368763 23259697

[9] Bursac N (2014). Cardiac fibroblasts in pressure overload hypertrophy: the enemy within?. J Clin Invest.

[10] Hewitson TD, Holt SG, Smith ER (2015). Animal models to study links between cardiovascular disease and renal failure and their relevance to human pathology. Front Immunol.

[11] Seong E, Saunders TL, Stewart CL, Burmeister M (2004). To knockout in 129 or in C57BL/6: that is the question. Trends Genet.

[12] Kren S, Hostetter TH (1999). The course of the remnant kidney model in mice. Kidney Int.

[13] Yokozawa T, Oura H, Okada T (1982). Metabolic effects of dietary purine in rats. J Nutr Sci Vitaminol (Tokyo).

[14] Jia T, Olauson H, Lindberg K, Amin R, Edvardsson K, Lindholm B, Andersson G, Wernerson A, Sabbagh Y, Schiavi S, Larsson TE (2013). A novel model of adenine-induced tubulointerstitial nephropathy in mice. BMC Nephrol.

[15] Chen J, Kieswich JE, Chiazza F, Moyes AJ, Gobbetti T, Purvis GS, Salvatori DC, Patel NS, Perretti M, Hobbs AJ, Collino M, Yaqoob MM, Thiemermann C (2017). IκB kinase inhibitor attenuates sepsis-induced cardiac dysfunction in CKD. J Am Soc Nephrol.

[16] Caton PW, Nayuni NK, Kieswich J, Khan NQ, Yaqoob MM, Corder R (2010). Metformin suppresses hepatic gluconeogenesisthrough induction of SIRT1 and GCN5. J Endocrinol.

[17] Jensen EC (2013). Quantitative analysis of histological staining and fluorescence using ImageJ. Anat Rec (Hoboken).

[18] Narula J, Haider N, Virmani R, DiSalvo TG, Kolodgie FD, Hajjar RJ, Schmidt U, Semigran MJ, Dec GW, Khaw BA (1996). Apoptosis in myocytesin end-stage heart failure. N Engl J Med.

[19] Olivetti G, Abbi R, Quaini F, Kajstura J, Cheng W, Nitahara JA, Quaini E, Di Loreto C, Beltrami CA, Krajewski S, Reed JC, Anversa P (1997). Apoptosis in the failing human heart. N Engl J Med.

[20] Ezekowitz RA, Austyn J, Stahl PD, Gordon S (1981). Surface properties of bacillus Calmette-Guerin-activated mouse macrophages. Reduced expression of mannose-specific endocytosis, Fc receptors, and antigen F4/80 accompanies induction of Ia. J Exp Med.

[21] Chanutin A, Ferris EB (1932). Experimental renal insufficiency produced by partial nephrectomy. Arch Intern Med.

[22] Vaneerdeweg W, Buyssens N, De Winne T, Sebrechts M, Babloyan A, Arakelian S (1992). A standardized surgical technique to obtain a stable and reproducible chronic renal failure model in dogs. Eur Surg Es.

[23] Liu S, Kompa AR, Kumfu S, Nishijima F, Kelly DJ, Krum H, Wang BH (2013). Subtotal nephrectomy accelerates pathological cardiac remodelling post-myocardial infarction:implications for cardiorenal syndrome. Int J Cardiol.

[24] van Dokkum RP, Eijkelkamp WB, Kluppel AC, Henning RH, van Goor H, Citgez M, Windt WA, van Veldhuisen DJ, de Graeff PA, de Zeeuw D (2004). Myocardial infarction enhances progressive renal damage in an experimental model for cardio-renal interaction. J Am Soc Nephrol.

[25] Bongartz LG, Joles JA, Verhaar MC, Cramer MJ, Goldschmeding R, Tilburgs C, Gaillard CA, Doevendans PA, Braam B (2012). Subtotal nephrectomy plus coronary ligation leads to more pronounced damage in both organs than either nephrectomy or coronary ligation. Am J Physiol Heart Circ Physiol.

[26] Price PA, Roublick AM, Williamson MK (2006). Artery calcification in uremic rats is increased by a low protein diet and prevented by treatment with ibandronate. Kidney Int.

[27] Koeda T, Wakaki K, Koizumi F, Yokozawa T, Oura H (1988). Early changes of proximal tubules in the kidney of adenine-ingesting rats, with special reference to biochemical and electron microscopic studies. Nippon Jinzo Gakkai Shi.

[28] Katsumata K, Kusano K, Hirata M, Tsunemi K, Nagano N, Burke SK, Fukushima N (2003). Sevelamer hydrochloride prevents ectopic calcification and renal osteodystrophy in chronic renal failure rats. Kidney Int.

[29] Rambausek M, Ritz E, Mall G, Mehls O, Katus H (1985). Myocardial hypertrophy in rats with renal insufficiency. Kidney Int.

[30] Pluim BM, Zwinderman AH, van der Laarse A, van der Wall EE (2000). The athlete’s heart. A meta-analysis of cardiac structure and function. Circulation.

[31] Borlaug BA (2014). The pathophysiology of heart failure with preserved ejection fraction. Nat Rev Cardiol.

[32] Byrne CJ, McCafferty K, Kieswich J, Harwood S, Andrikopoulos P, Raftery M, Thiemermann C, Yaqoob MM (2012). Ischemic conditioning protects the uremic heart in a rodent model of myocardial infarction. Circulation.

[33] Matsui T, Nagoshi T, Rosenzweig A (2003). Akt and PI 3-kinase signaling in cardiomyocyte hypertrophy and survival. Cell Cycle.

[34] Matsui T, Tao J, del Monte F, Lee KH, Li L, Picard M, Force TL, Franke TF, Hajjar RJ, Rosenzweig A (2001). Akt activation preserves cardiac function and prevents injury after transient cardiac ischemia in vivo. Circulation.

[35] Haq S, Choukroun G, Lim H, Tymitz KM, del Monte F, Gwathmey J, Grazette L, Michael A, Hajjar R, Force T, Molkentin JD (2001). Differential activation of signal transduction pathways in human hearts with hypertrophy versus advanced heart failure. Circulation.

[36] Knowlton AA, Connelly CM, Romo GM, Mamuya W, Apstein CS, Brecher P (1992). Rapid expression of fibronectin in the rabbit heart after myocardial infarction with and without reperfusion. J Clin Invest.

[37] Tomasek JJ, Gabbiani G, Hinz B, Chaponnier C, Brown RA (2002). Myofibroblasts and mechano-regulation of connective tissue remodelling. at Rev Mol Cell Biol.

[38] Shiojima I, Aikawa M, Suzuki J, Yazaki Y, Nagai R (1999). Embryonic smooth muscle myosin heavy chain SMemb is expressed in pressure-overloaded cardiac fibroblasts. Jpn Heart J.

[39] Conway B, Hughes J (2012). Cellular orchestrators of renal fibrosis. QJM.

[40] Lan A, Zhang J, Xiao Z, Peng X, Qi Y, Du J (2014). Akt2 is involved in loss of epithelial cells and renal fibrosis following unilateral ureteral obstruction. PLoS One.

[41] Kattla JJ, Carew RM, Heljic M, Godson C, Brazil DP (2008). Protein kinase B/AKT activity is involved in renal TGF-beta1-driven epithelial-mesenchymal transition in vitro and in vivo. Am J Physiol Renal Physiol.

[42] Furuya S, Chappell GA, Iwata Y, Uehara T, Kato Y, Kono H, Bataller R, Rusyn I (2016). A mouse model of alcoholic liver fibrosis-associated acute kidney injury identifies key molecular pathways. Toxicol Appl Pharmacol.

[43] Suzuki K, Nakagawa K, Yamamoto T, Miyazawa T, Kimura F, Kamei M (2015). Carbon tetrachloride-induced hepatic and renal damages in rat: inhibitory effects of cacaopolyphenol. Biosci Biotechnol Biochem.

[44] Yang HC, Zuo Y, Fogo AB (2010). Models of chronic kidney disease. Drug Discov Today Dis Models.

[45] Fukuda S, Kopple JD (1980). Chronic uremia syndrome in dogs induced with uranyl nitrate. Nephron.

[46] Okuda S, Y O, Tsuruda H, Onoyama K, Fujimi S, Fujishima M (1986). Adriamycin-induced nephropathy as a model of chronic progressive glomerular disease. Kidney Int.

