# Supplementary material for: A novel model of reno-cardiac syndrome in the C57BL/ 6 mouse strain
Source: BMC Nephrol. 2018 Dec 4;19:346. doi: 10.1186/s12882-018-1155-3 (PMC6278034; doi:10.1186/s12882-018-1155-3)
Supplement: Supplementary file 1 — Adenine diet composition. (PDF 1108 kb) [file 12882_2018_1155_MOESM1_ESM.pdf]

Special Diets Services

Rat and Mouse No.1 Maintenance

Pelleted, Expanded and Expanded Ground

SUITABLE SPECIES AND APPLICATIONS

Rats and mice for long and short term maintenance.

BENEFITS

- High quality human food grade soya bean concentrate provides a less variable source of protein.
- Low protein level promotes longer life expectancy, reducing obesity and associated problems in the aged animal.
- Low nutrient levels reduce the risk of undesirable side-effects in toxicity trials being masked.

FEEDING GUIDE

Ad-lib feeding is recommended.

AVAILABLE AS

Diet	Form	Product Code
Standard		
RM1 (P)	*9.5mm Pelleted*	*801151*
RM1 (E)	*Expanded*	*801002*
SQC		
RM1 (E) SQC	*Expanded*	*811002*
RM1 (E) FG SQC	*Expanded Ground*	*811004*

- All diets are available irradiated and are available in a range of packaging.
- All Standard diets are available with full analysis on request.

INGREDIENTS

Wheat, Barley, Wheatfeed, De-hulled Extracted Toasted Soya, Soya Protein Concentrate, Macro Minerals, Soya Oil, Whey Powder, Amino Acids, Vitamins, Micro Minerals.

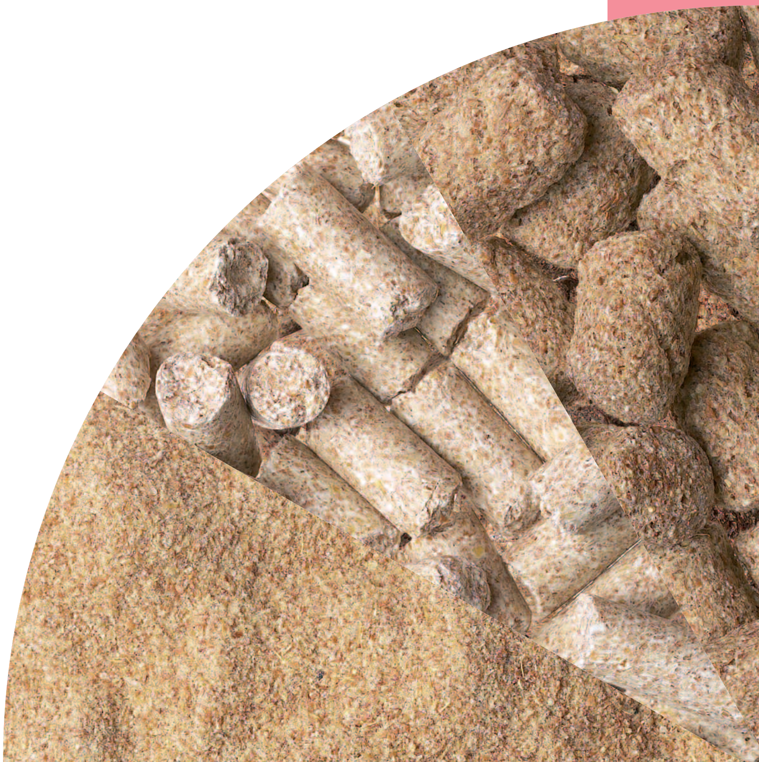

LBS-Biotech email: sales@lbs-biotech.com

Calculated Analysis

NUTRIENTS		Total	Supp (9)
**Proximate Analysis**			
Moisture (1)	%	10.00	
Crude Oil	%	2.71	
Crude Protein	%	14.38	
Crude Fibre	%	4.65	
Ash	%	6.00	
Nitrogen Free Extract	%	61.73	
**Digestibility Co-Efficients (7)**			
Digestible Crude Oil	%	2.47	
Digestible Crude Protein	%	12.92	
**Carbohydrates, Fibre and Non Starch Polysaccharides (NSP)**			
Total Dietary Fibre	%	17.05	
Pectin	%	1.52	
Hemicellulose	%	10.17	
Cellulose	%	4.32	
Lignin	%	1.68	
Starch	%	44.97	
Sugar	%	4.05	
**Energy (5)**			
Gross Energy	MJ/kg	14.74	
Digestible Energy (15)	MJ/kg	11.90	
Metabolisable Energy (15)	MJ/kg	10.74	
Atwater Fuel Energy (AFE)(8)	MJ/kg	13.75	
AFE from Oil	%	7.42	
AFE from Protein	%	17.49	
AFE from Carbohydrate	%	75.09	
**Fatty Acids**			
Saturated Fatty Acids			
C12:0 Lauric	%	0.02	
C14:0 Myristic	%	0.14	
C16:0 Palmitic	%	0.31	
C18:0 Stearic	%	0.04	
Monounsaturated Fatty Acids			
C14:1 Myristoleic	%	0.02	
C16:1 Palmitoleic	%	0.09	
C18:1 Oleic	%	0.77	
Polyunsaturated Fatty Acids			
C18:2(ω6) Linoleic	%	0.69	
C18:3(ω3) Linolenic	%	0.06	
C20:4(ω6) Arachidonic	%	0.13	
C22:5(ω3) Clupanodonic	%		
**Amino Acids**			
Arginine	%	0.91	
Lysine (6)	%	0.66	0.07
Methionine	%	0.22	0.04
Cystine	%	0.24	
Tryptophan	%	0.18	
Histidine	%	0.35	
Threonine	%	0.49	
Isoleucine	%	0.54	
Leucine	%	0.98	
Phenylalanine	%	0.66	
Valine	%	0.69	
Tyrosine	%	0.49	
Taurine	%		
Glycine	%	1.11	
Aspartic Acid	%	0.67	

NUTRIENTS		Total	Supp (9)
Glutamic Acid	%	3.17	
Proline	%	1.20	
Serine	%	0.56	
Hydroxyproline	%		
Hydroxylysine	%		
Alanine	%	0.16	
**Macro Minerals**			
Calcium	%	0.73	0.63
Total Phosphorus	%	0.52	0.04
Phytate Phosphorus	%	0.24	
Available Phosphorus	%	0.28	0.04
Sodium	%	0.25	0.19
Chloride	%	0.38	0.32
Potassium	%	0.67	
Magnesium	%	0.23	
**Micro Minerals**			
Iron	mg/kg	159.30	82.50
Copper	mg/kg	11.50	1.94
Manganese	mg/kg	72.44	19.22
Zinc	mg/kg	35.75	
Cobalt	µg/kg	634.10	550.00
Iodine	µg/kg	1202.69	1085.00
Selenium	µg/kg	298.99	100.00
Fluorine	mg/kg	10.49	
**Vitamins**			
β-Carotene (2)	mg/kg	0.16	
Retinol (2)	µg/kg	2566.38	2400.00
Vitamin A (2)	iu/kg	8554.27	8000.00
Cholecalciferol (3)	µg/kg	15.54	15.00
Vitamin D (3)	iu/kg	621.70	600.00
α-Tocopherol (4)	mg/kg	76.45	56.82
Vitamin E (4)	iu/kg	84.10	62.50
Vitamin B_1 (Thiamine)	mg/kg	8.58	1.96
Vitamin B_2 (Riboflavin)	mg/kg	4.33	2.94
Vitamin B_6 (Pyridoxine)	mg/kg	4.81	0.98
Vitamin B_{12} (Cyanocobalamine)	µg/kg	7.49	6.00
Vitamin C (Ascorbic Acid)	mg/kg	2.59	
Vitamin K (Menadione)	mg/kg	10.17	9.36
Folic Acid (Vitamin B_9)	mg/kg	0.79	
Nicotinic Acid (Vitamin PP) (6)	mg/kg	61.32	2.45
Pantothenic Acid (Vitamin $B_{3/5}$)	mg/kg	20.17	5.80
Choline (Vitamin $B_{4/7}$)	mg/kg	1080.14	366.60
Inositol	mg/kg	2369.59	
Biotin (Vitamin H) (6)	µg/kg	277.13	

Notes

1. All values are calculated using a moisture basis of 10%.
   Typical moisture levels will range between 9.5 - 11.5%.
2. a. Vitamin A includes Retinol and the Retinol equivalents of β-carotene
   b. Retinol includes the Retinol equivalents of β-Carotene.
   c. 0.48 µg Retinol = 1 µg β-carotene = 1.6 iu Vitamin A activity
   d. 1 µg Retinol = 3.33* iu Vitamin A activity
   e. 1 iu Vitamin A = 0.3 µg Retinol = 0.6 µg β-carotene
   f. The standard analysis for Vitamin A does not detect β-carotene
3. 1µg Cholecalciferol (D_3) = 40.0 iu Vitamin D
4. 1 mg all-*rac*-α-tocopherol = 1.1 iu Vitamin E activity
   1 mg all-*rac*-α-tocopherol acetate = 1.0 iu Vitamin E activity
5. 1 MJ = 239.23 Kcalories = 239.23 Calories = 239,230 calories
6. These nutrients coming from natural raw materials such as cereals may have low availabilities due to the interactions with other compounds.
7. Based on in-vitro digestibility analysis.
8. AF Energy = Atwater Fuel Energy = ((CO%/100)*9000)+((CP%/100)*4000)+((NFE%/100)*4000)/239.23
9. Supplemented nutrients from manufactured and mined sources.
15. Calculated.
