# Supplementary figures and images for: A novel model of reno-cardiac syndrome in the C57BL/ 6 mouse strain
Source: BMC Nephrol. 2018 Dec 4;19:346. doi: 10.1186/s12882-018-1155-3 (PMC6278034; doi:10.1186/s12882-018-1155-3)

[a]

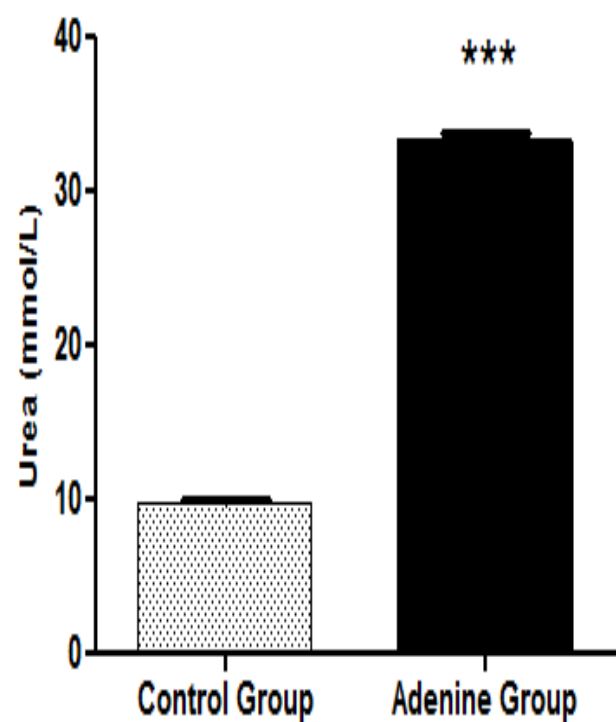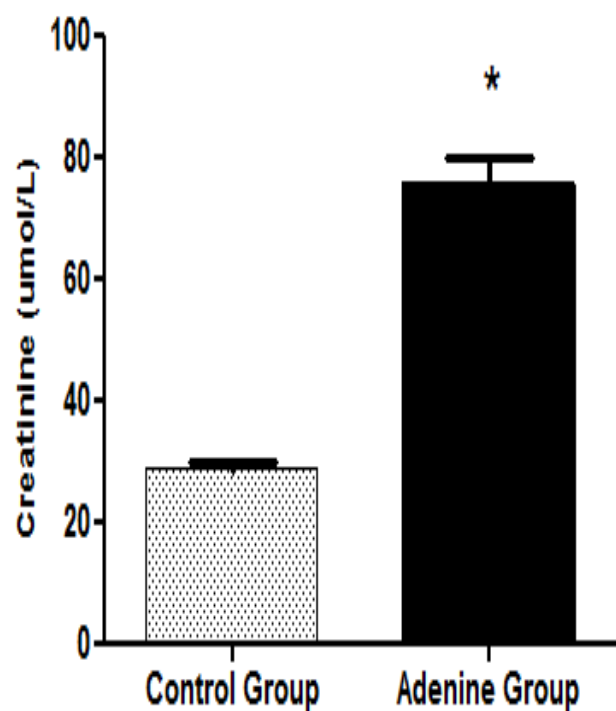

[b]

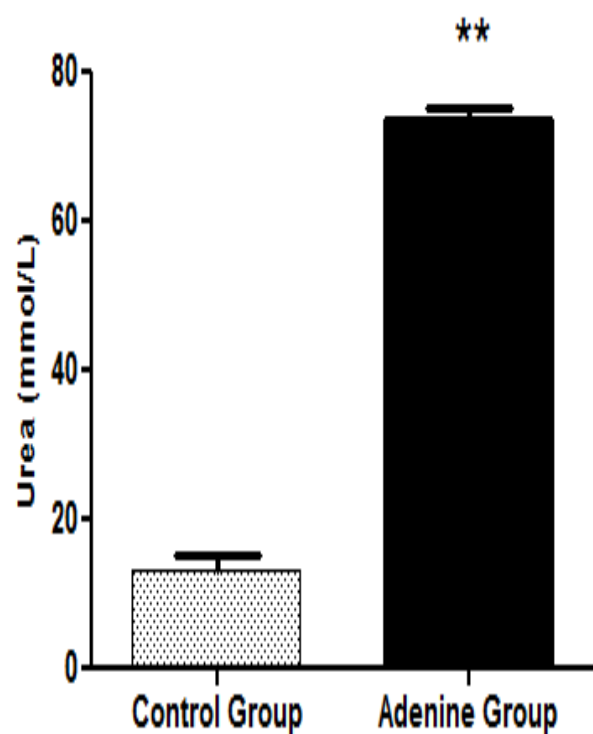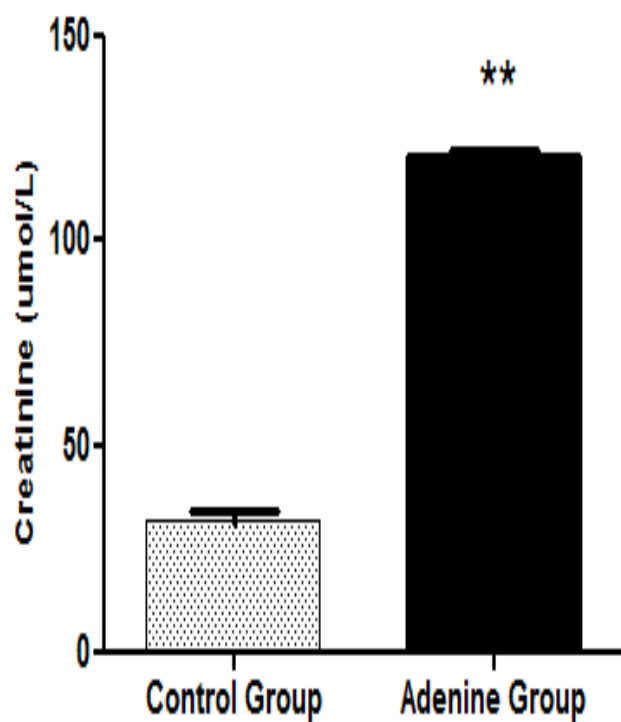

Supplement: Supplementary file 2 — Weeks 12 [a] and 16 [b] serum biochemistry data. Two animals from each group were sacrificed at weeks 12 and 16 in order to determine the progression of uraemia. Serum urea and creatinine were determined by an enzymatic method (IDEXX Bioresearch, Ludwigsberg, Germany). (PDF 95 kb) [file 12882_2018_1155_MOESM2_ESM.pdf]

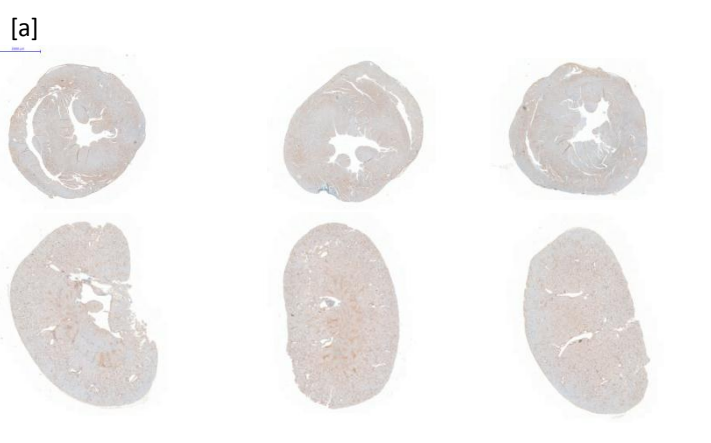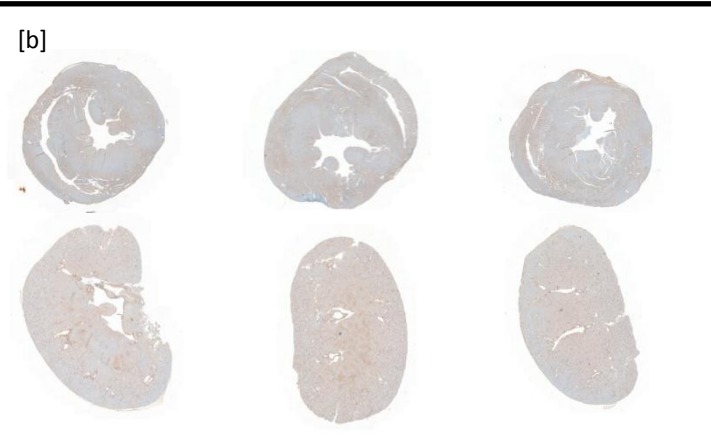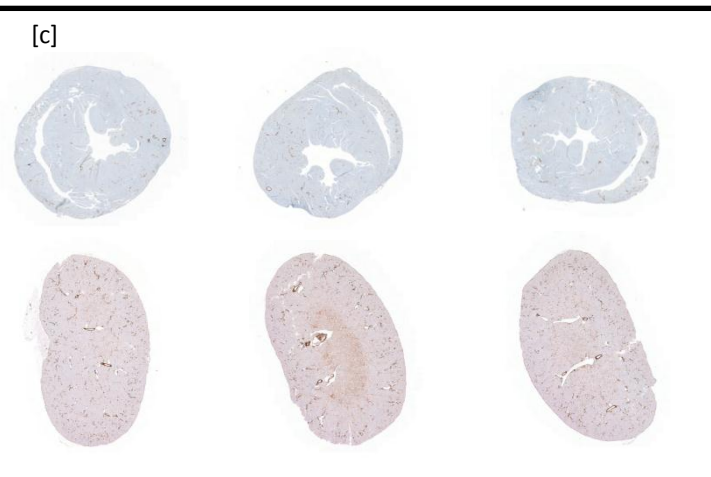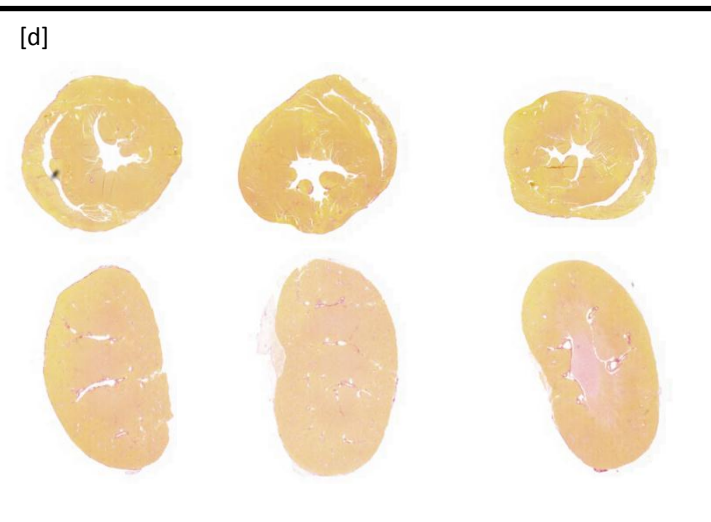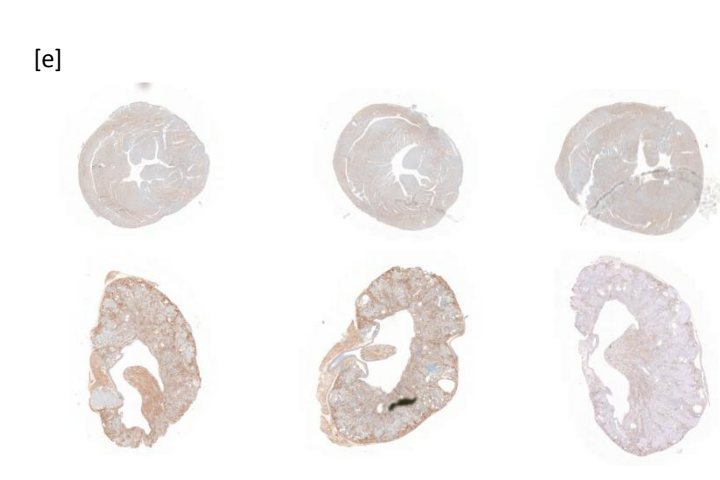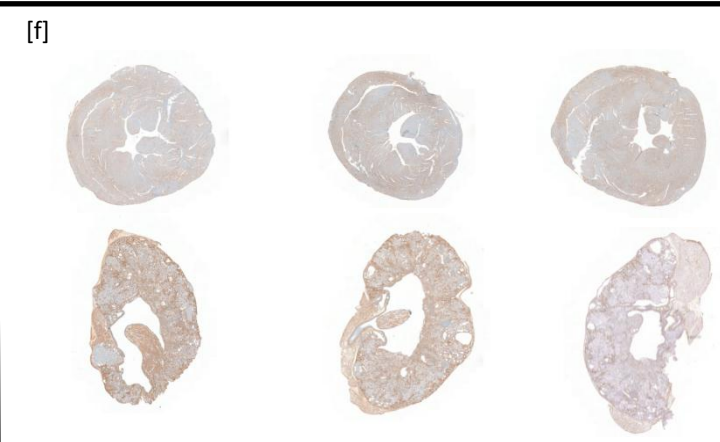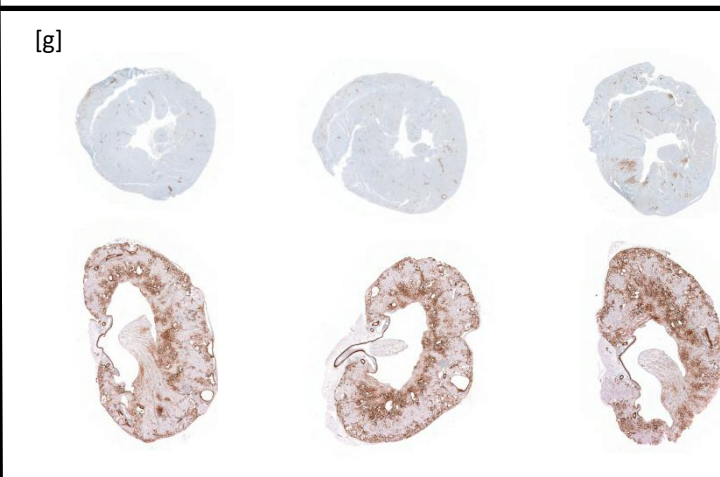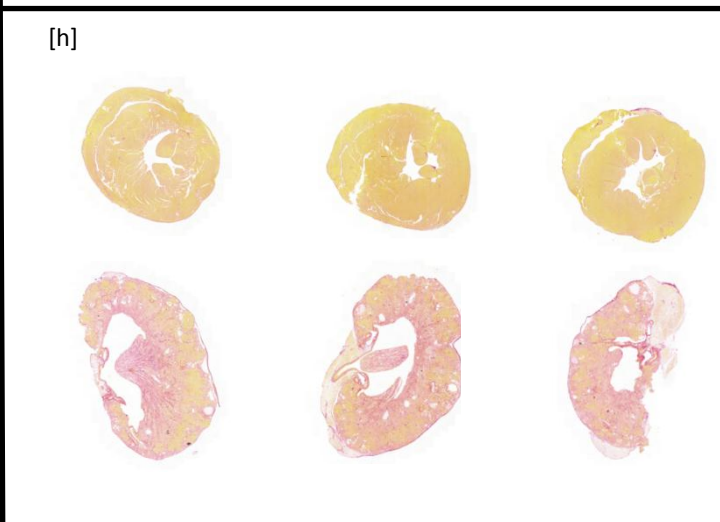

Supplement: Supplementary file 5 — All slides, collagen 1, (control [a], adenine [e]). Collagen 3, (control [b], adenine [f]). α-SMA (control [c], adenine [g]). Sirius red, (control [d], adenine [h]). (PDF 370 kb) [file 12882_2018_1155_MOESM5_ESM.pdf]

a

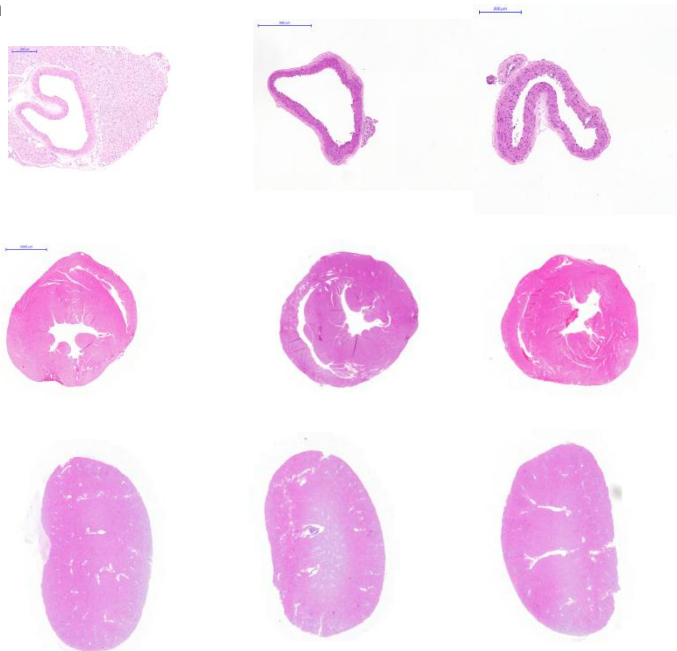

d

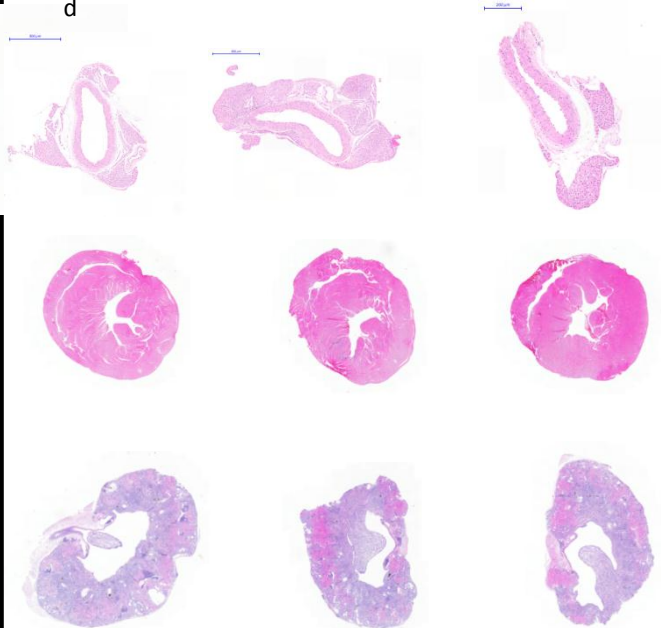

b

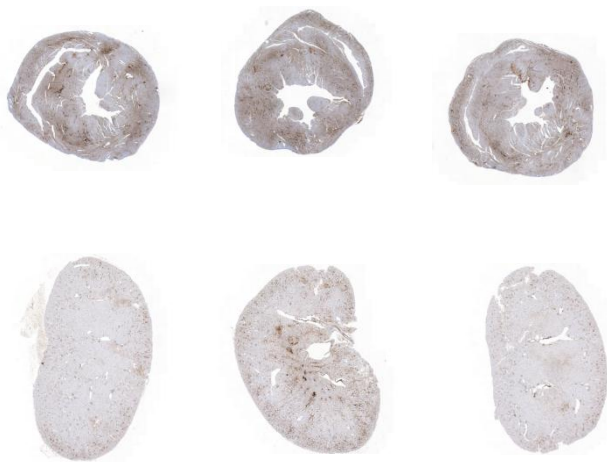

e

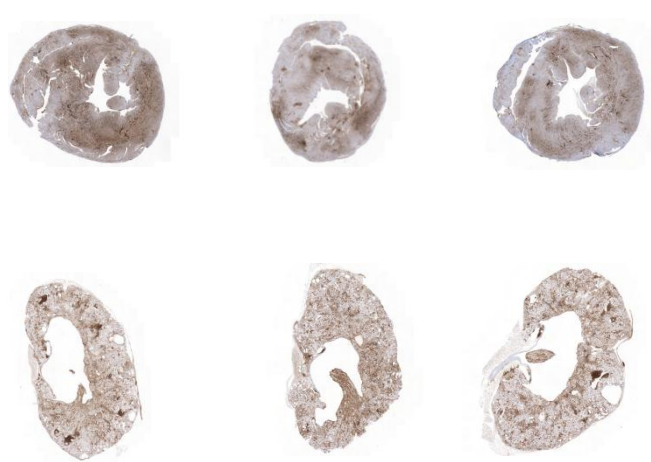

c

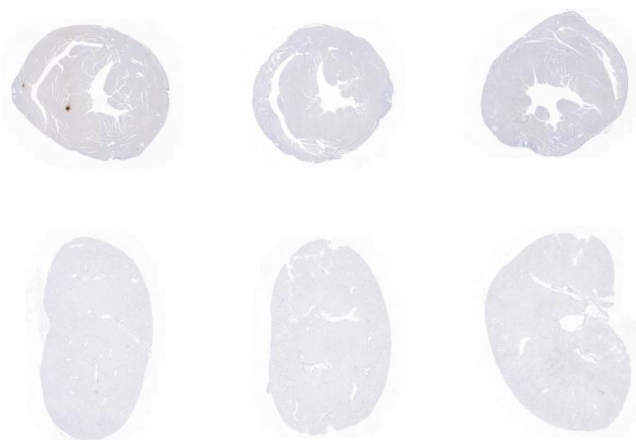

f

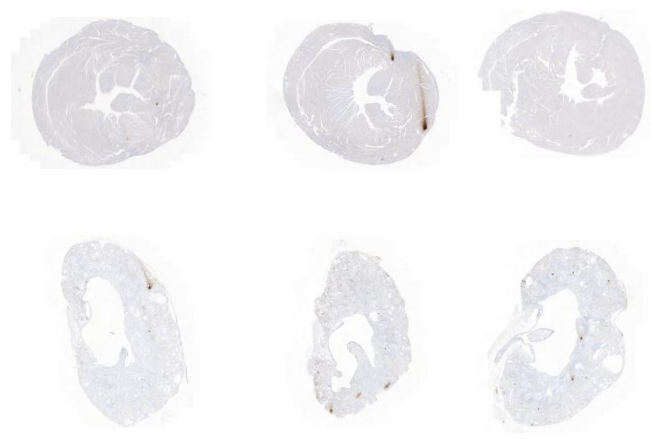

Supplement: Supplementary file 6 — All slides, H and E, (control [a], adenine [d]). CD45, (control [b], adenine [e]). F4/80, (control [c], adenine [f]). (PDF 323 kb) [file 12882_2018_1155_MOESM6_ESM.pdf]

[a]

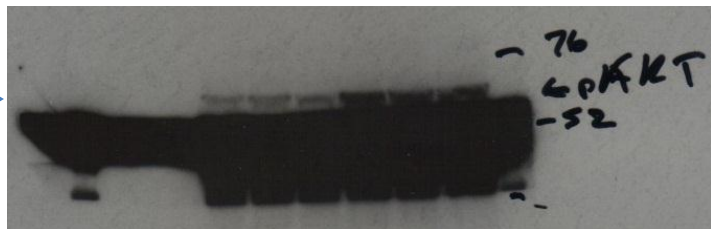

[b]

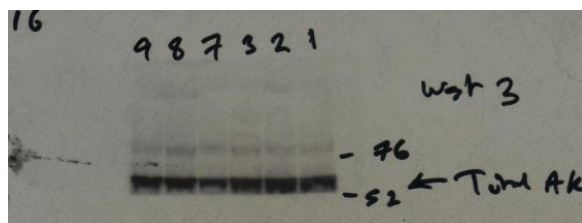

[c]

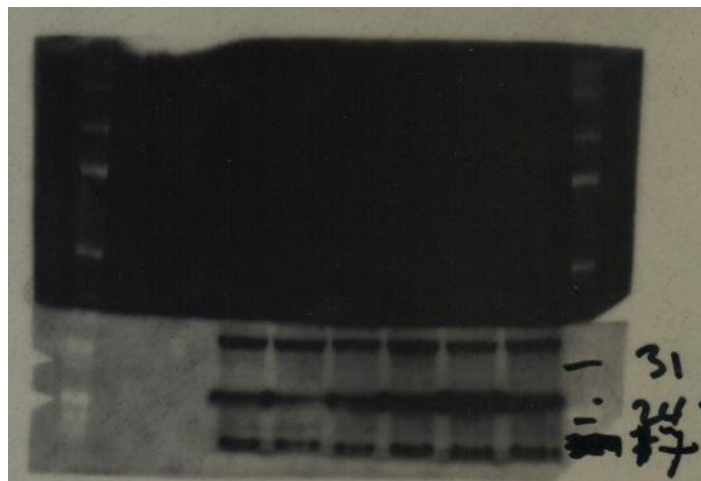

[d]

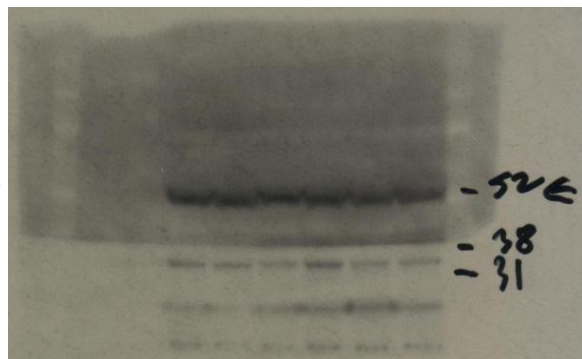

[e]

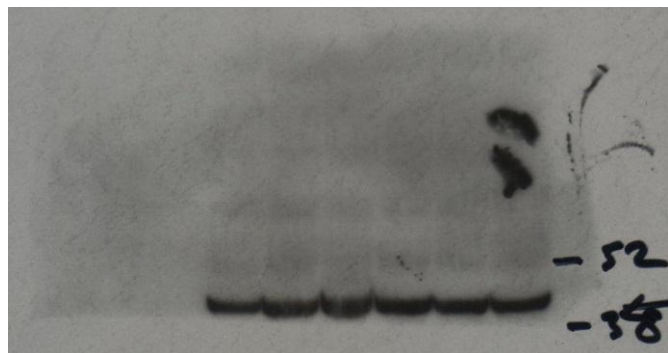

[f]

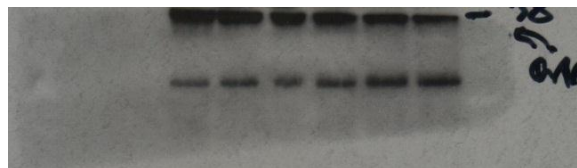

[g]

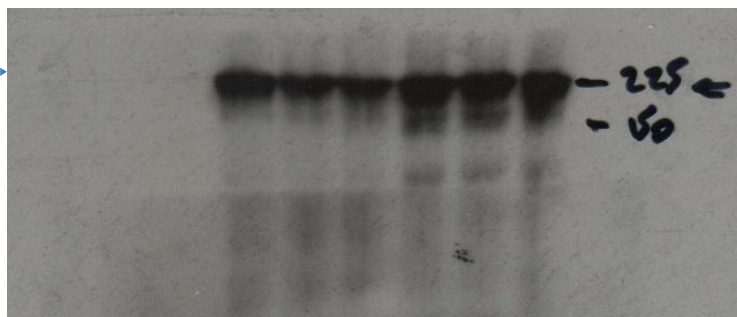

[h]

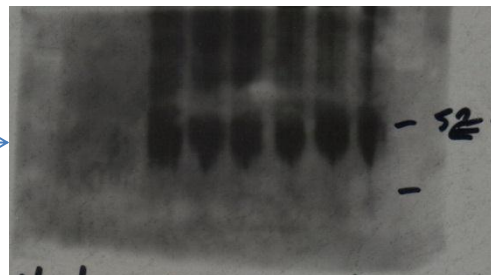

Supplement: Supplementary file 7 — Uncropped Western blots: hearts p AKT/total AKT ([a]/[b]), hearts caspase 3/α-tubulin ([c]/[d]), hearts α-SMA/Gapdh ([e]/[f]), hearts fibronectin/α-tubulin ([g]/[h]). (PDF 207 kb) [file 12882_2018_1155_MOESM7_ESM.pdf]

[a]

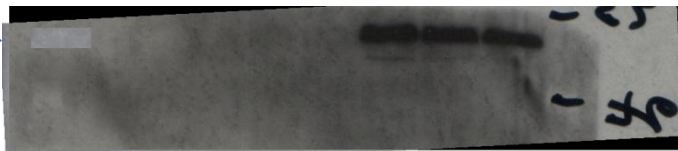

[b]

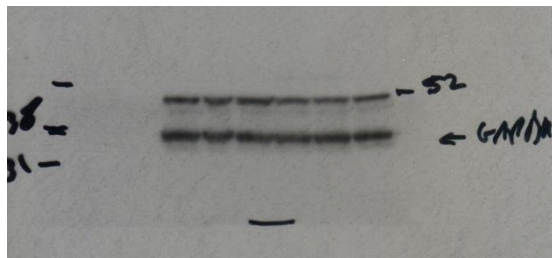

[c]

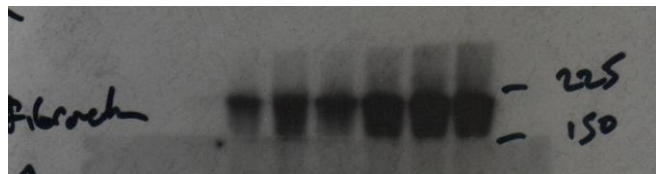

[d]

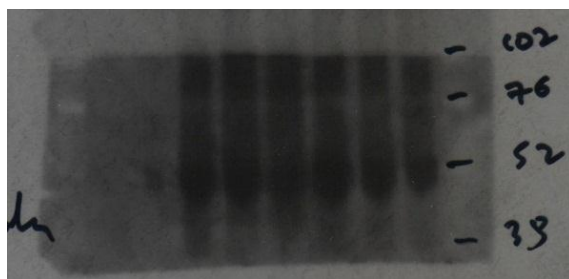

[e]

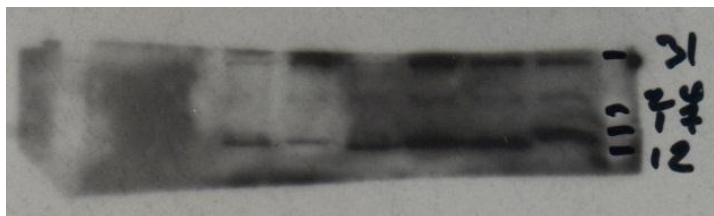

[f]

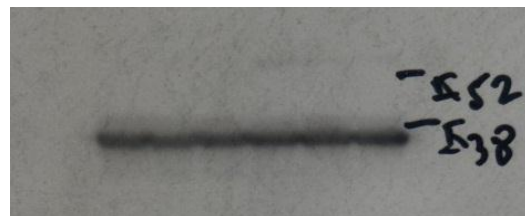

[g]

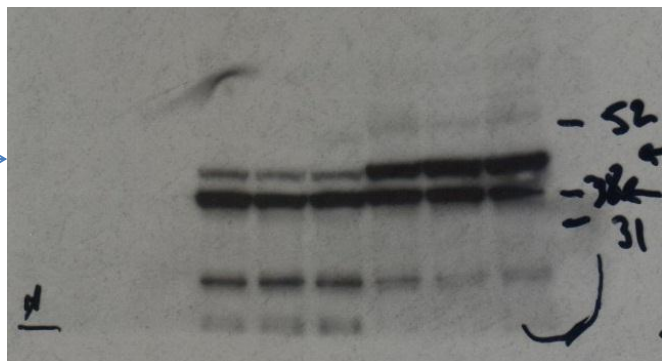

[h]

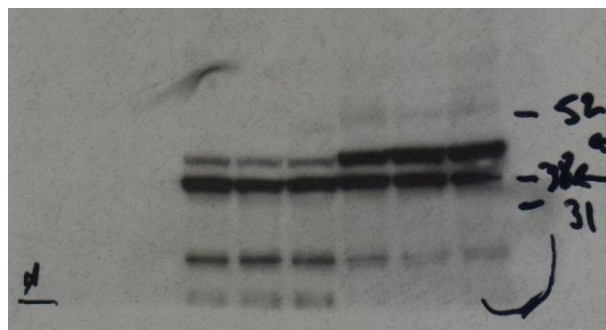

Supplement: Supplementary file 8 — Uncropped Western blots: kidneys total AKT/Gapdh ([a]/[b]), kidneys fibronectin/α-tubulin, ([c]/[d]), kidneys caspase 3/Gapdh ([e]/[f]), kidneys α-SMA/Gapdh ([g]/[h]). (PDF 181 kb) [file 12882_2018_1155_MOESM8_ESM.pdf]
