# Supplementary material for: A novel model of reno-cardiac syndrome in the C57BL/ 6 mouse strain
Source: BMC Nephrol. 2018 Dec 4;19:346. doi: 10.1186/s12882-018-1155-3 (PMC6278034; doi:10.1186/s12882-018-1155-3)
Supplement: Supplementary file 3 — Table S3. Biochemistry measurements methods. (PDF 168 kb) [file 12882_2018_1155_MOESM3_ESM.pdf]

Parameter	Method of measurement
Creatinine	Jaffe alkaline picrate
Urea	Enzymatic UV test
Sodium	Indirect potentiometry
Potassium	Indirect potentiometry
Calcium	Cresolphthalein complex
Inorganic phosphorous	Ammonium-Phosphomolybdate-complex, UV-Test
